# Complete Genome Sequence of *Edwardsiella hoshinae* ATCC 35051

**DOI:** 10.1128/genomeA.01605-16

**Published:** 2017-02-09

**Authors:** Stephen R. Reichley, Geoffrey C. Waldbieser, Mark L. Lawrence, Matt J. Griffin

**Affiliations:** a College of Veterinary Medicine, Mississippi State University, Starkville, Mississippi, USA; b Aquatic Research and Diagnostic Laboratory, Thad Cochran National Warmwater Aquaculture Center, Stoneville, Mississippi, USA; c USDA, ARS Warmwater Aquaculture Research Unit, Thad Cochran National Warmwater Aquaculture Center, Stoneville, Mississippi, USA

## Abstract

*Edwardsiella hoshinae* is a Gram-negative facultative anaerobe that has primarily been isolated from avians and reptiles. We report here the complete and annotated genome sequence of an isolate from a monitor lizard (*Varanus* sp.), which contains a chromosome of 3,811,650 bp and no plasmids.

## GENOME ANNOUNCEMENT

The *Edwardsiella* genus was first recognized in the 1960s based on the new species *Edwardsiella tarda*, constituting a group of enteric Gram-negative bacteria (1). Shortly thereafter, a group of *Edwardsiella* strains demonstrating unique biochemical processes were isolated in Japan. These strains formed a cluster distinct from other *E. tarda* isolates based on biochemical, physiological, and morphological characters and were considered a biotype of *E. tarda* (2). Further investigations using biochemical and DNA relatedness methods identified this distinct clade as a novel species, designated *Edwardsiella hoshinae* (3). Relative to other *Edwardsiella* spp., there is limited information regarding *E. hoshinae*. This bacterium has been isolated from birds, reptiles, water, and human feces; however, its role as a human pathogen has not been established, and it is not considered a zoonotic agent (3–6). At present, strain ATCC 35051 is the only published complete genome sequence for *Edwardsiella hoshinae*.

Genomic DNA was sequenced using Pacific Biosciences (PacBio) technology to a depth of 25× genome coverage (96.5 Mb). Reads ≥8,000 bp were error corrected with shorter PacBio reads and assembled using Canu version 1.0 (7). Illumina sequences (109× coverage, minimum depth of 11) were mapped to the PacBio assembly using BWA version 0.7.10-r789 (8), and base errors and insertions/deletions were corrected iteratively using Pilon version 1.16 (9) until no further base corrections were made automatically. Overlapping sequence was identified, and the linear contig was circularized and relinearized at a position 1 million bases distant from the original position of circularization. Illumina and PacBio sequences were realigned and visualized using the Integrated Genome Viewer (10) for validation of contiguity and manual correction of assembly errors.

The circularized and completed genome sequence was submitted to the National Center for Biotechnology Information (NCBI) Prokaryotic Genome Annotation Pipeline (PGAP) for annotation and submission to GenBank. The genome sequence was also submitted for Rapid Annotations using Subsystems Technology (RAST) analysis (11, 12) employing the Glimmer option. Average nucleotide identities (ANI) (13) and digital DNA-DNA hybridization (dDDH) (14) estimations were determined using online calculators (ANI, http://enve-omics.ce.gatech.edu/ani/; dDDH, http://ggdc.dsmz.de/distcalc2.php).

This *E. hoshinae* genome consists of one circular chromosome with 3,811,650 bp (56.9% G+C content). PGAP annotation predicted 3,401 genes encoding 3,204 proteins and 101 tRNAs. RNAmmer (15) predicted nine rRNA operons. RAST analysis predicted 497 subsystems with 3,526 coding sequences and 128 RNAs. The complete genome of ATCC 35051 shares an ANI of 87.4% (dDDH, 35%) with *E. tarda* isolate FL95-01 (16); 83.1% (dDDH, 25%) with *E. anguillarum* isolates LADL05-105 (17), EA181011 (18), and ET080813 (19, 20); 83.0% (dDDH, 25%) with *E. piscicida* isolates S11-285 (21) and C07-087 (22); and 82.6% (dDDH, 24%) with *E. ictaluri* isolate 93-146 (23). We detected no plasmids in ATCC 35051. The complete annotated genome of *Edwardsiella hoshinae* will be useful for the proper determination of taxonomic affiliation of future *Edwardsiella* species genomes, as well as investigations into host-pathogen interactions.

### Accession number(s).

The complete genome sequence for *Edwardsiella hoshinae* isolate ATCC 35051 has been deposited in GenBank under accession no. CP016043.

## References

[1] Ewing WH, McWhorter AC, Escobar MR, Lubin AH 1965 *Edwardsiella*, a new genus of *Enterobacteriaceae* based on a new species, *E. tarda*. Int J Syst Evol Microbiol 15:33–38. doi:10.1099/00207713-15-1-33.

[2] Johnson R, Colwell RR, Sakazaki R, Tamura K 1975 Numerical taxonomy study of the *Enterobacteriacae*. Int J Syst Bacteriol 25:12–37. doi:10.1099/00207713-25-1-12.

[3] Grimont PAD, Grimont F, Richard C, Sakazaki R 1980 *Edwardsiella hoshinae*, a new species of *Enterobacteriaceae*. Curr Microbiol 4:347–351. doi:10.1007/BF02605375.

[4] Farmer JJ, McWhorter AC 1984 Genus X. *Edwardsiella*. *In* Krieg NR, Holt JG (ed), Bergey’s manual of systematic bacteriology. Lippincott Williams & Wilkins, Baltimore, MD.

[5] Singh IM, Singh S, Mills-Robertson F, McMurphy MA, Applegate RD, Crupper SS 2004 Antibiotic susceptibility of *Edwardsiella hoshinae* isolated from northern bobwhite quail (*Colinus virginianus*). Vet Rec 155:29–29. doi:10.1136/vr.155.1.29. 15264489

[6] Singh BR, Singh V, Ebibeni N, Singh RK 2013 Antimicrobial and herbal drug resistance in enteric bacteria isolated from faecal droppings of common house lizard/gecko (*Hemidactylus frenatus*). Int J Microbiol 2013:340848. doi:10.1155/2013/340848. 24223595 PMC3800600

[7] Berlin K, Koren S, Chin CS, Drake JP, Landolin JM, Phillippy AM 2015 Assembling large genomes with single-molecule sequencing and locality-sensitive hashing. Nat Biotechnol 33:623–630. doi:10.1038/nbt.3238. 26006009

[8] Li H 2013 Aligning sequence reads, clone sequences and assembly contigs with BWA-MEM. arXiv arXiv:1303.3997v2.

[9] Walker BJ, Abeel T, Shea T, Priest M, Abouelliel A, Sakthikumar S, Cuomo CA, Zeng Q, Wortman J, Young SK, Earl AM 2014 Pilon: an integrated tool for comprehensive microbial variant detection and genome assembly improvement. PLoS One 9:e112963. doi:10.1371/journal.pone.0112963. 25409509 PMC4237348

[10] Thorvaldsdóttir H, Robinson JT, Mesirov JP 2013 Integrative genomics viewer (IGV): high-performance genomics data visualization and exploration. Brief Bioinform 14:178–192. doi:10.1093/bib/bbs017. 22517427 PMC3603213

[11] Aziz RK, Bartels D, Best AA, DeJongh M, Disz T, Edwards RA, Formsma K, Gerdes S, Glass EM, Kubal M, Meyer F, Olsen GJ, Olson R, Osterman AL, Overbeek RA, McNeil LK, Paarmann D, Paczian T, Parrello B, Pusch GD, Reich C, Stevens R, Vassieva O, Vonstein V, Wilke A, Zagnitko O 2008 The RAST server: rapid annotations using subsystems technology. BMC Genomics 9:75. doi:10.1186/1471-2164-9-75. 18261238 PMC2265698

[12] Overbeek R, Olson R, Pusch GD, Olsen GJ, Davis JJ, Disz T, Edwards RA, Gerdes S, Parrello B, Shukla M, Vonstein V, Wattam AR, Xia F, Stevens R 2014 The SEED and the Rapid annotation of microbial genomes using subsystems technology (RAST). Nucleic Acids Res 42:D206–D214. doi:10.1093/nar/gkt1226. 24293654 PMC3965101

[13] Goris J, Konstantinidis KT, Klappenbach JA, Coenye T, Vandamme P, Tiedje JM 2007 DNA-DNA hybridization values and their relationship to whole-genome sequence similarities. Int J Syst Evol Microbiol 57:81–91. doi:10.1099/ijs.0.64483-0. 17220447

[14] Auch AF, von Jan M, Klenk HP, Göker M 2010 Digital DNA-DNA hybridization for microbial species delineation by means of genome-to-genome sequence comparison. Stand Genomic Sci 2:117–134. doi:10.4056/sigs.531120. 21304684 PMC3035253

[15] Lagesen K, Hallin P, Rødland EA, Staerfeldt H-H, Rognes T, Ussery DW 2007 RNAmmer: consistent and rapid annotation of ribosomal RNA genes. Nucleic Acids Res 35:3100–3108. doi:10.1093/nar/gkm160. 17452365 PMC1888812

[16] Reichley SR, Waldbieser GC, Tekedar HC, Lawrence ML, Griffin MJ 2015 Complete genome sequence of *Edwardsiella tarda* isolate FL95-01, recovered from channel catfish. Genome Announc 3(3):e00682-15. doi:10.1128/genomeA.00682-15. 26112788 PMC4481286

[17] Reichley SR, Waldbieser GC, Lawrence ML, Griffin MJ 2015 Complete genome sequence of an *Edwardsiella piscicida*-like species, recovered from tilapia in the United States. Genome Announc 3(5):e01004-15. doi:10.1128/genomeA.01004-15. 26337892 PMC4559741

[18] Reichley SR, Waldbieser GC, Ucko M, Colorni A, Dubytska L, Thune RL, Lawrence ML, Griffin MJ 2015 Complete genome sequence of an *Edwardsiella piscicida*-like species isolated from diseased grouper in Israel. Genome Announc 3(4):e00829-15. doi:10.1128/genomeA.00829-15. 26205870 PMC4513164

[19] Yang M, Lv Y, Xiao J, Wu H, Zheng H, Liu Q, Zhang Y, Wang Q 2012 *Edwardsiella* comparative phylogenomics reveal the new intra/inter-species taxonomic relationships, virulence evolution and niche adaptation mechanisms. PLoS One 7:e36987. doi:10.1371/journal.pone.0036987. 22590641 PMC3349661

[20] Shao S, Lai Q, Liu Q, Wu H, Xiao J, Shao Z, Wang Q, Zhang Y 2015 Phylogenomics characterization of a highly virulent *Edwardsiella* strain ET080813(T) encoding two distinct T3SS and three T6SS gene clusters: Propose a novel species as *Edwardsiella anguillarum* sp. nov. Syst Appl Microbiol 38:36–47. doi:10.1016/j.syapm.2014.10.008. 25466920

[21] Reichley SR, Waldbieser GC, Tekedar HC, Lawrence ML, Griffin MJ 2016 Complete genome sequence of *Edwardsiella piscicida* Isolate S11-285 recovered from channel catfish (*Ictalurus punctatus*) in Mississippi, USA. Genome Announc 4(6):e01259-16. doi:10.1128/genomeA.01259-16. 27881536 PMC5122678

[22] Tekedar HC, Karsi A, Williams ML, Vamenta S, Banes MM, Duke M, Scheffler BE, Lawrence ML 2013 Complete genome sequence of channel catfish gastrointestinal septicemia isolate *Edwardsiella tarda* C07-087. Genome Announc 1(6):e00959-13. doi:10.1128/genomeA.00959-13. 24265493 PMC3837174

[23] Williams ML, Gillaspy AF, Dyer DW, Thune RL, Waldbieser GC, Schuster SC, Gipson J, Zaitshik J, Landry C, Banes MM, Lawrence ML 2012 Genome sequence of *Edwardsiella ictaluri* 93–146, a strain associated with a natural channel catfish outbreak of enteric septicemia of catfish. J Bacteriol 194:740–741. doi:10.1128/JB.06522-11. 22247535 PMC3264089

